# Long-Term Health-Related Quality of Life Following Survival of Acute Respiratory Distress Syndrome and Extracorporeal Membrane Oxygenation Due to COVID-19

**DOI:** 10.3390/jcm14103358

**Published:** 2025-05-12

**Authors:** Martina Hermann, Rebecca Filipsky, Nils Bukowski, Gernot Gerger, Alexander Hermann, Katharina Krenn, Anna Teufel, Oliver Kimberger, Daniel Laxar, Mathias Maleczek, Eva Schaden, Marion Wiegele, Harald Willschke, Akos Tiboldi

**Affiliations:** 1 Department of Anaesthesia, Clinical Division of General Anaesthesia and Intensive Care Medicine, Intensive Care Medicine and Pain Medicine, Medical University of Vienna, 1090 Vienna, Austria; 2 Ludwig Boltzmann Institute Digital Health and Patient Safety, 1180 Vienna, Austria; 3 Intensive Care Unit 13i2, Department of Medicine I, Medical University of Vienna, 1090 Vienna, Austria

**Keywords:** acute respiratory distress syndrome, extracorporeal membrane oxygenation, COVID-19, prospective cohort study, telephone interview, health-related quality of life, post-intensive care syndrome

## Abstract

**Background:** Patients suffering from severe COVID-19 often develop acute respiratory distress syndrome (ARDS), necessitating intensive care unit (ICU) and extracorporeal membrane oxygenation (ECMO). Survivors frequently experience negative impacts on their health-related quality of life. These individuals may experience a range of symptoms and may require extended hospitalization and rehabilitation. The objective of this prospective cohort study was to assess the long-term health-related quality of life in intensive care survivors of COVID-19-related ARDS who received ECMO therapy, >18 months after their ICU discharge. **Methods:** The health-related quality of life of COVID-19 survivors who had received extracorporeal membrane oxygenation was evaluated using an augmented version of the Short-Form Health Survey-36, >18 months after their ICU discharge. The outcomes were compared to preexisting data from a meta-analysis analyzing patients with non-COVID-19 ARDS and ECMO therapy. **Results:** Of the 43 eligible patients (mean age 52 ± 9.5 years), 18 patients (46.2%) responded to the written invitation and were included in this study. The four subscales of the Short-Form Health Survey-36 survey, performed via telephone interview, that showed the most severe limitations (points) were role limitation due to physical problems (37.5), emotional problems (47.9), social functioning (38.1), and general health (49.2). The general health, energy/fatigue (vitality), and physical functioning significantly correlated with higher age (*p* = 0.004, *p* = 0.003, and *p* = 0.05, respectively). A longer duration of extracorporeal membrane oxygenation was positively associated with an improved energy/fatigue ratio (vitality) and emotional well-being (*p* = 0.04 and *p* = 0.02, respectively). Compared to survivors of non-COVID-19 ARDS treated with ECMO, the survivors in our cohort scored significantly lower on social functioning, physical functioning, and general health (*p* < 0.01, *p* = 0.02, *p* < 0.01). **Conclusions:** Patients who have recovered from intensive care treatment for COVID-19-related ARDS and have received ECMO therapy continue to experience more severe impairments in their physical, mental, and cognitive health-related quality of life. A longer ECMO duration may improve outcomes in this selected patient population.

## 1. Introduction

Currently, most patients suffering from acute coronavirus disease 2019 (COVID-19) are oligo- or asymptomatic, although even in these cases, chronic symptoms may occur in the long term. Depending on the predominant variant, up to 20% of patients with acute infection develop severe symptoms requiring hospitalization and 5–8% of the total infected population requires intensive care unit (ICU) treatment [1,2,3,4,5]. Recent evidence suggests that pre-infection COVID-19 vaccination may reduce the ICU admission risk and mitigate select long-term symptoms such as fatigue, anxiety, and palpitations in ICU survivors, although studies show mixed results on the overall quality of life and have not yet comprehensively assessed post-intensive care syndrome (PICS) specifically [6,7,8,9]. The use of extracorporeal membrane oxygenation (ECMO) therapy in severe COVID-19-related acute respiratory distress syndrome (ARDS) is recommended [10,11,12,13,14]. Advances in medical care have led to more patients surviving intensive care, increasing the number of patients at risk of post-intensive care syndrome (PICS). PICS describes a new or worsening decrease in physical, mental, and/or cognitive functioning that occurs after ICU treatment [15]. Unfortunately, the risk stratification, prediction models, and treatment of PICS are still limited [16]. Older age, female sex, history of mental health problems, duration of sedation, neuromuscular blocking agents, disease severity, muscle weakness, hypoxia, negative ICU experience, and delirium are risk factors for PICS [17]. Also, COVID-19 may cause long-lasting physical and mental effects. The corresponding syndrome is named post-acute COVID-19 syndrome (PCS) [18]. Many symptoms of PCS are similar to those of PICS, including shortness of breath, joint or chronic pain, brain fog, depression, fatigue, sleep disturbance, and motor or sensory dysfunction [19]. The Center of Disease Control defines PCS, also known as long COVID, as a “lack of return to a usual state of health following acute COVID-19 illness” [20].

An indicator of PICS severity is the subjectively perceived health-related quality of life (HRQOL) [21]. The HRQOL is a patient-reported outcome that reflects the physical state and mental health based on patient perception. It is most commonly assessed using either the Short-Form Health Survey-36 (SF-36) [22] or EuroQOL-5D (EQ-5D) [23] questionnaire. Most conventionally treated (without ECMO) COVID-19 ICU survivors seem to have PICS symptoms after hospital discharge [24], with prevalence rates as high as 90% among ICU survivors [25]. However, data on the long-term HRQOL outcomes of patients who survived severe COVID-19-related ARDS, including ECMO therapy, are scarce [26,27,28] and, particularly, prospective evaluations using patient-centered outcome measures are rare. Early work by Hodgson et al. [29] and more recent systematic reviews [30,31] show that ECMO survivors generally experience reduced physical functioning and quality of life, often accompanied by psychological symptoms such as anxiety and PTSD. Still, outcomes vary: some cohorts show a comparable or even better HRQOL than conventionally treated ARDS survivors [32,33], and recovery can improve over time. Notably, in this vulnerable population, greater knowledge of both physical and cognitive post-intensive care sequelae is warranted in order to deepen our understanding of the significant interval from discharge to “recovery”. Therefore, we performed a comprehensive assessment of the HRQOL of hospital survivors following ICU treatment, including ECMO for COVID-19-related ARDS more than 18 months after ICU discharge.

## 2. Materials and Methods

### 2.1. Study Design

This prospective observational cohort study took place between June 2022 and December 2022. This study was approved by the Ethics Committee of the Medical University of Vienna (EK 2440/2020) and was performed in accordance with the Declaration of Helsinki [34] and the applicable laws and regulations in Austria. Study design, data handling, and reporting followed the STROBE [35] guidelines to ensure maximum research quality.

### 2.2. Study Sites

Intensive care treatment was provided by the Vienna General Hospital/Medical University of Vienna, Department of Anaesthesia, Intensive Care Medicine and Pain Medicine (ICU 9D, 13C1, 13C2, 13C3), Department of Medicine I (ICU 13i2), and telephone interviews were conducted by the Ludwig Boltzmann Institute Digital Health and Patient Safety, Vienna, Austria.

### 2.3. Inclusion Criteria

**Figure 1:**
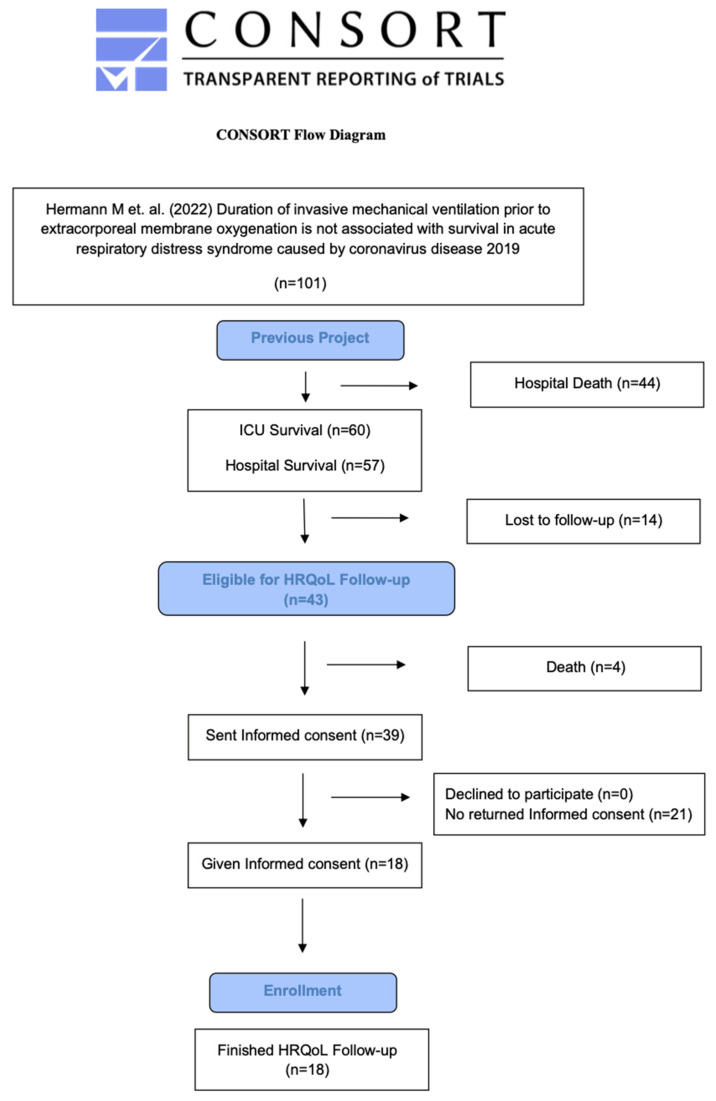
Consort Flow Diagram. ICU = intensive care unit; HRQOL = health-related quality of life; ref. [36].

Patients aged ≥18 years >18 months after ICU discharge who were treated with ECMO due to confirmed COVID-19-related ARDS between January 2020 until May 2021 in the above-mentioned ICUs. Figure 1 shows the CONSORT Flow Diagram. ICU survivors were informed of the study’s objectives, invited to participate, and requested to provide informed consent via postal communication. Written informed consent was obtained from all study participants before the start of the study. After informed written consent was obtained, participants were contacted via telephone for a one-time structured interview >18 months after ICU discharge. All interviews were conducted by the same trained physician using a standardized protocol.

### 2.4. Exclusion Criteria

Only participants who provided informed consent were included in the study. No other exclusion criteria were applied.

### 2.5. Endpoints, Questionnaire and Interview

To uncover the effects on HRQOL in patients treated with ECMO for severe COVID-19-related ARDS > 18 months after ICU discharge, we assessed HRQOL using a standardized SF-36 questionnaire [18]. The SF-36 questionnaire is subdivided into eight health sub-concepts (*physical functioning*, *role limitation physical*, *role limitation emotional*, *energy*/*fatigue* (*vitality*), *emotional well-being, social functioning, pain*, and *general health*). It ranges from 0 to 100 points for each health concept, with a higher score indicating a more favorable health state.

We included additional questions regarding demographic data. The study was conducted through telephone interviews. All interviews were conducted by the same physician (MH) throughout the study period to avoid examiner bias.

Further, we aimed to uncover the relationship between HRQOL variables and clinical parameters at the time of treatment (medication, ICU data, and socio-demographic variables), as this could help to further understand the factors potentially influencing HRQOL outcomes.

### 2.6. Data Collection

Patient identification and data collection were conducted using the database from our previously published study [36]. The Simplified Acute Physiology Score III (SAPS III) [37] was evaluated on ICU admission. Study contact data, including postal addresses and telephone numbers, were collected from the patient data management system’s routine documentation (ICCA©, Philips, Amsterdam, The Netherlands). Data processing and storage were performed on a server at the Department of Anaesthesia, Intensive Care Medicine and Pain Medicine.

### 2.7. Statistical Methods

First, we provide a descriptive overview of the data as means (standard deviation) and sample sizes for demographic data, ICU data, and comorbidities. The primary endpoint was whether ECMO treatment for patients with severe ARDS due to COVID-19 influenced HRQOL > 18 months after ICU discharge. To uncover changes in HRQOL (SF-36 scores) in our sample, we compared our HRQOL estimates to the pooled weighted mean HRQOL scores (Table 1) of patients suffering from non-COVID-19-related ARDS undergoing ECMO provided in the meta-analyses by Turgeon et al. [38]. Due to violations of parametric test assumptions, we conducted a non-parametric one-sample Wilcoxon signed-rank test against the pooled mean score from the meta-analyses. Effect sizes for the Wilcoxon signed-rank test are provided by rank biserial correlations *r*. All statistical analyses were based on an alpha level of 0.05. No corrections for multiple testing were applied, as all tests should be considered exploratory.

In the second analysis step, we correlated HRQOL (SF-36 scores) outcomes with age, sex, BMI, medications, and ICU data using non-parametric Spearman’s rank correlational tests. The analyses were conducted using R and R-Studio [39,40]. A correlational plot was produced using the ggstatsplot package 0.13.1 [41].

Paperpal was used to support language refinement during manuscript drafting. All content generated or suggested by the tool was thoroughly reviewed and verified by the authors. No AI tools were used for data analysis, interpretation, or generation of scientific content.

## 3. Results

### 3.1. Descriptive Data

In total, 18 patients (46.2%) out of 43 eligible patients consented to the study and completed the interviews. The patients were treated with ECMO for COVID-19-related ARDS between January 2020 and May 2021. The mean age was 52 [SD ± 9.5] years, nine patients [50%] were men, and the patients had a mean BMI of 30 [SD ± 5] kg/m^2^. The patients had a mean ECMO duration of 21.4 [SD ± 12.2] days, a mean invasive mechanical ventilation (IMV) pre-ECMO duration of 8.93 [SD ± 7.96] days, a mean ICU LOS of 41.61 [SD ± 21.6] days, and a mean SAPS III at ICU admission of 66.35 [SD ± 7.87]. Regarding the social aspects of life, eight patients (44%) had resumed work at the time of assessment, and three patients (21%, n = 14) retired early due to ICU treatment and a poor HRQOL. The demographic data and detailed information are presented in Table 2.

### 3.2. HRQOL (SF-36 Scores)

**Figure 2:**
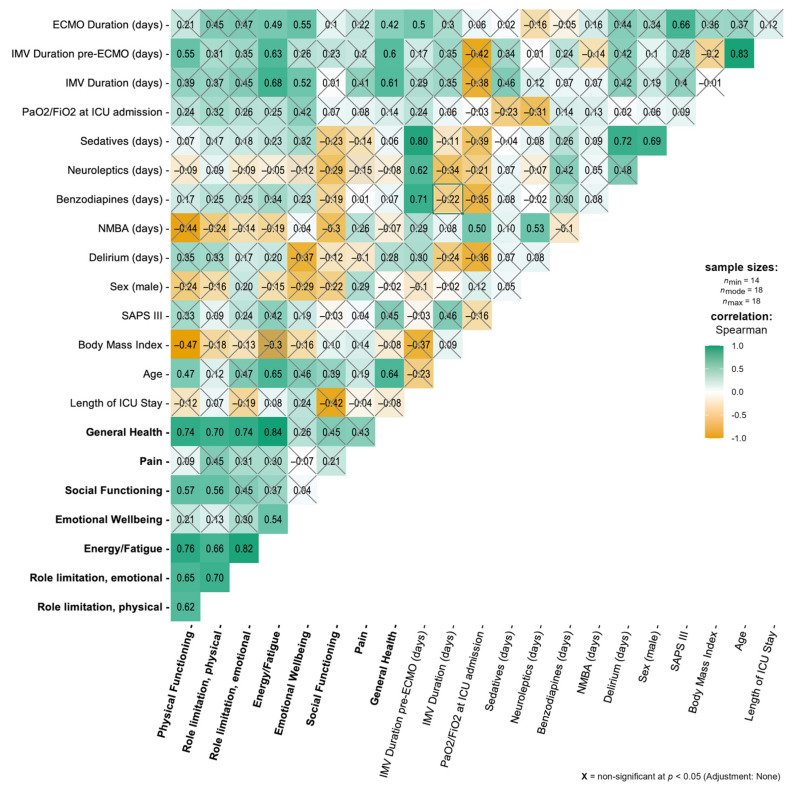
Correlations for demographics, medications, ICU data, and SF-36 subscales > 18 months after ICU discharge. Values represent Spearman’s rho (r). HRQOL (SF-36 concepts) is in bold font. ECMO, extracorporeal membrane oxygenation; IMV, invasive mechanical ventilation; PaO_2_/FiO_2_, ratio of arterial oxygen partial pressure to fractional inspired oxygen; ICU, intensive care unit; NMBA, neuromuscular blocking agents; SAPS III, Simplified Acute Physiology Score III.

The mean score of *physical functioning* was 62.22 [SD ± 32.95], and the subscale regarding *bodily pain* had a mean score of 75.14 [SD ± 32.95]. *Role limitations* are expressed by two scores: The score for *role limitation* due to *emotional problems* was 47.92 [SD ± 50.14], and *role limitation* due to *physical issues* had a mean score of 37.50 [SD ± 48.70]. The subscale of *emotional well-being* had a mean score of 70.89 [SD ± 23.83], the result for *social functioning* was 38.14 [SD ± 41.45], the mean score for *energy*/*fatigue ratio* (*vitality*) was 51.02 [SD ± 33.97], and the *general health* perception had a mean score of 49.24 [SD ± 30.03]. Table 1 provides a descriptive overview of the HRQOL scores. Figure 2 additionally provides the inter-construct correlations of the HRQOL (bold letters).

### 3.3. HRQOL Outcomes Compared to a Non-COVID-19 ECMO Survivor Post-ICU Patients

Our patients showed several significant reductions in the HRQOL variables compared to the non-COVID-19 ECMO patients [37]. Survivors of severe ARDS due to COVID-19 on ECMO scored significantly lower on *social functioning* (V_Wilcoxon_ = 23.00, *p* < 0.01, *r* = −0.73), *physical functioning* (V_Wilcoxon_ = 32.00, *p* = 0.02, *r* = −0.63), and *general health* (V_Wilcoxon_ = 61.00, *p* < 0.01, *r* = −0.29). No significant differences for the remaining sub-concepts were uncovered for *role limitations due to physical problems* (V_Wilcoxon_ = 94.00, *p* = 0.72, *r* = 0.10), *role limitations due to emotional problems* (V_Wilcoxon_ = 35.00, *p* = 0.09, *r* = −0.49), *pain* (V_Wilcoxon_ = 91.00, *p* = 0.82, *r* = 0.06), *energy*/*fatigue* (*vitality*) (V_Wilcoxon_ = 75.00, *p* = 0.66, *r* = 0.12), and *emotional well-being* (V_Wilcoxon_ = 100.00, *p* = 0.54, *r* = 0.17). Please see Table 1.

#### 3.3.1. Demographic Data

The mean age was 52 [SD ± 9.5] years; a higher age was positively correlated with the subscale *energy*/*fatigue* (*vitality*) (*p* = 0.003; *r* = 0.65), *general health* (*p* = 0.004; *r* = 0.64), and *physical functioning* (*p* = 0.05; *r* = 0.47). A higher BMI (mean BMI 30 [SD ± 5] kg/m^2^) was negatively correlated with *physical functioning* (*p* = 0.05; *r* = −0.47). Obesity and male sex were significantly associated with more days on NMBA (*p* = 0.04; *r* = 0.5; *p* = 0.02; *r* = 0.53). The number of days on NMBA was negatively correlated with *physical functioning* (*p* = 0.07; *r* = −0.44). A longer ICU length of stay was associated with lower *social functioning* scores (*p* = 0.11; *r* = −0.42). These correlations are shown in Figure 2 and Supplemental Table S1. The demographic data of the patients without a response at the HRQOL follow-up are shown in Supplementary Table S2.

#### 3.3.2. ICU-Related Data

A longer ECMO duration (mean ECMO duration 21.4 [SD ± 12.2] days) was positively correlated with *better energy*/*fatigue* (*vitality*) (*r* = 0.49; *p* = 0.04) and *emotional well-being* (*p* = 0.02; *r* = 0.55). Unsurprisingly, a longer ECMO duration was significantly correlated with a longer ICU length of stay (*p* = 0.05; *r* = 0.5) and more days on sedatives (*p* < 0.001; *r* = 0.8), neuroleptics (*p* = 0.01; *r* = 0.62), and benzodiazepines (*p* = 0.002; *r* = 0.71). Furthermore, a longer IMV duration (mean IMV duration 25.2 [SD ±11.83] days) was positively correlated with *general health and energy*/*fatigue* (*vitality*) (*p* = 0.007; *r* = 0.61, *p* = 0.002; *r* = 0.68). In addition, a longer pre-ECMO IMV duration (mean pre-ECMO IMV duration 8.93 [SD ± 7.96] days) was positively correlated with *general health* (*p* = 0.009; *r* = 0.6), *energy*/*fatigue* (*vitality*) (*p* = 0.005; *r* = 0.63), *and physical functioning* (*p* = 0.019; *r* = 0.55). A *lower physical role limitation* score was associated with further impairment in *energy*/*fatigue* (*vitality*), *general health*, *social functioning*, and *role limitation due to emotional problems*. These correlations are shown in Figure 2.

The detailed outcomes of the correlational tests can be found in Supplementary Table S1.

## 4. Discussion

In this study, we examined the long-term HRQOL after ECMO therapy for COVID-19-related ARDS post-ICU discharge. The four subscales of the SF-36 survey that showed the most severe limitations were *role limitation due to physical problems* (37.5), *role limitations due to emotional problems* (47.9), *social functioning* (38.1), and *general health* (49.2). *General health*, *energy*/*fatigue* (*vitality*), and *physical functioning* were significantly correlated with age (*p* = 0.004, *p* = 0.003, and *p* = 0.05, respectively).

The post-COVID-19 condition is characterized by a range of persistent symptoms, including breathlessness, reduced exercise tolerance, fatigue, frailty, muscle pain, brain fog, sleep disturbance, depression, and anxiety that persist for months [43,44,45,46,47,48]. Often, there are serious effects on the mental health of patients surviving hospitalization owing to COVID-19. The prevalence of posttraumatic stress disorder (PTSD) symptoms in conventionally treated COVID-19 patients a few months after discharge from the hospital varies in the literature and has been reported with an average of approximately 15% [49,50,51]. PTSD symptoms typically emerge within the first few months after ICU discharge, with prevalence peaking between 3 and 9 months and persisting in about 20% of patients at one year after ICU discharge [52,53,54]. Our findings of ongoing mental health impairment >18 months post-discharge may reflect this chronic burden and the presence of late-onset or unresolved PTSD symptoms. Other factors affecting mental health, such as depression, anxiety, loneliness, and insomnia, are equally prevalent among patients who survive hospitalization due to conventional treatment for COVID-19 [55,56,57,58,59].

The long-term quality of life in patients surviving severe ARDS treated with ECMO in the ICU is of paramount interest. However, it is equally vital to focus on achieving substantial recovery and a satisfactory quality of life after ICU treatment in the medium and long term. The growing number of ECMO applications has made it increasingly crucial to address potential lasting impairments and develop strategies to alleviate both physical and mental consequences [60]. In the context of a post-COVID-19 condition coinciding with a recent ICU discharge, the risk of daily life impairments may be particularly high.

In this study, we also addressed the potential impact of selection bias introduced by non-responders to follow-up. Among the 43 eligible patients, 18 (46.2%) participated in the study. This suggests that our population might overrepresent patients with either better recovery or fewer psychological impairments. While this limitation must be acknowledged, our response rate remains comparable to those reported in similar post-ICU HRQOL studies [38].

In our study population, an extended duration of ECMO (mean 21.4 [SD ± 12.2] days) was positively associated with better HRQOL scores in the domains of *energy/fatigue (vitality)* and *emotional well-being* (*p* = 0.04 and *p* = 0.02). In contrast, Rilinger et al. [32] identified no significant association between the ECMO duration and HRQoL in a large cohort of non-COVID-19 ARDS survivors. This discrepancy may be partially attributed to differences in statistical methodologies, as their analysis utilized median splits, a technique known to diminish statistical power and obscure true correlations in continuous outcome variables [61]. Furthermore, the relatively short ECMO durations reported in other studies, such as Wang et al. [62], who documented a mean ECMO run of merely 6 days, may constrain the ability to detect duration-related effects. Conversely, the broader range and longer average duration in our cohort may have enhanced sensitivity in identifying associations with post-ICU recovery. Our findings are supported by Dreier et al. [63], who observed that prolonged ECMO support (median 28 [IQR 13;64] days) in COVID-19 patients was linked to favorable clinical outcomes. Similarly, Shah et al. [64] reported satisfactory functional status and an acceptable HRQOL in select patients following extended ECMO treatment for COVID-19 ARDS. However, not all evidence concurs with this perspective: Rai et al. [65] found that longer ECMO durations were associated with impaired physical functioning and increased difficulty in performing daily activities. These mixed results highlight the complexity of post-ECMO recovery, likely influenced by factors such as sedation practices, neuromuscular blockade, and access to rehabilitation. Moreover, it is possible that our observed correlation is influenced by survivorship bias, whereby only those patients who tolerated prolonged ECMO and survived were available for follow-up, thus skewing outcomes toward more favorable recovery profiles. Nevertheless, our findings should be interpreted with caution. The substantially larger sample size of Rilinger et al.’s study [32] (n = 289) lends considerable weight to their null finding. In contrast, our analysis is based on a small, exploratory cohort (n = 18), which limits its statistical power and generalizability. The observed positive correlations, while noteworthy, may reflect specific patient selection or survival bias and should be validated in larger, prospective studies.

In our cohort, a longer ECMO duration correlated with a better HRQoL in selected domains, possibly reflecting reduced exposure to deep sedation or NMBAs. However, this remains speculative. Dreier et al. [63] reported favorable outcomes after prolonged ECMO but did not provide data on sedation or NMBA use. In contrast, Martínez et al. [66] found that higher cumulative doses of midazolam and NMBAs, along with prolonged mechanical ventilation and delirium, were linked to worse post-ICU outcomes in COVID-19 ARDS survivors. However, it should be noted that Martínez et al.’s cohort [66] did not include ECMO-treated patients, so direct comparison is limited. Our findings suggest that sedation-related risk factors may also be relevant in ECMO populations, although further targeted research is needed.

In our study population, *role limitations due to physical problems*, with a subscale of 37.5 describe a major restriction in daily life. Wang et al. [62] found no significant difference in the long-term SF-36 scores between non-COVID-19 ARDS survivors treated with ECMO and those treated conventionally, suggesting that ECMO itself did not contribute to HRQOL impairments in that population. In contrast, our COVID-19 ECMO cohort showed markedly lower scores, particularly in *social functioning* (38.14 vs. 71.42) and *role limitations due to emotional problems* (47.92 vs. 69.82), when compared to the non-COVID ECMO patients from a pooled meta-analysis [38]. These greater impairments may reflect pandemic-related factors—such as restricted family visitation, reduced patient–staff interaction due to protective equipment, and limited early mobilization—which likely compounded the psychological and physical burden of ICU care during COVID-19.

Together, all of these factors may contribute to PICS and PCS in patients with COVID-19 admitted to the ICU [67].

In view of the general risk factors for PICS and PCS, within our study population, age was significantly associated with higher *physical functioning*, *energy*/*fatigue* (*vitality*), *and general health* scores (mean age 52 [SD ± 9.5]). This is interesting, as Chommeloux et al. [28] discussed “advanced” age (median age 47 [IQR 40;55]) in patients with COVID-19-related ARDS as a relative contraindication for ECMO therapy, owing to physical and psychological impairments in these populations. The poor mental and physical HRQOL associated with PICS seemed to be, again, more related to COVID-19 than ICU treatment, including ECMO therapy, within the study population of Chommeloux et al. [28]. Similarly, Lee et al. [17] described general risk factors for PICS within a systematic review and meta-analysis, including higher age (34% prevalence of PICS in patients between 60 and 69 years), female sex, preexisting cognitive problems, disease severity, negative ICU experience, and delirium.

Another recently published study by Kosilek et al. [68] showed a “higher” age, (median 64 [IQR 53;72]), a longer ICU LOS (median 27 [IQR13;46]), and the IMV duration (median 12 [IQR 4;27]) as significantly associated risk factors for cognitive impairments in PICS in patients surviving ICU due to sepsis (without ECMO therapy) in Germany. These findings are the opposite of the results of our study, as the ICU LOS did not show any significant correlations with the HRQOL and PICS resp. PCS within our study population. A longer IMV duration (mean 25.2 [SD ±11.83]) within our study population was associated with better *energy*/*fatigue* (*vitality*), *emotional well-being*, *and general health* (*p* = 0.002, *p* = 0.028 and *p* = 0.007). In our own previous study [36] that included the same study population, we could show that a pre-ECMO IMV duration (>7 days) was not associated with survival in COVID-19 ARDS. In fact, a longer pre-ECMO IMV duration (mean pre-ECMO IMV duration 8.93 [SD ± 7.96] days) within the same study population was actually positively correlated with *general health* (*p* = 0.009; *r* = 0.6), *energy*/*fatigue* (*vitality*) (*p* = 0.005; *r* = 0.63), *and physical functioning* (*p* = 0.019; *r* = 0.55). These findings might be explainable due to a potentially lower IMV invasiveness over days.

We could demonstrate common preexisting illnesses in patients suffering from ARDS, such as obesity in 44%. A higher BMI (mean BMI 30 [SD ± 5] kg/m^2^) within our study population showed a significantly negative correlation with *physical functioning* (*p* = 0.05), probably explained by immobility due to more days on NMBA. These facts agree with the above-mentioned results reported by Martínez et al. [66]. Taboada et al. [69] compared patients with COVID-19 admitted to ICU (without ECMO therapy) and a normal ward. Within that study population, obesity did not lead to reductions in the HRQOL, potentially explained by there being only a third of mechanical ventilation days compared to our study population (8.5 [0;18] vs. 25.2 [±11.83] days) and, obviously, the reduced disease severity state.

Summarizing, within this study population, all patients continued to experience physical, mental, and/or cognitive restrictions for at least 18 months after ICU discharge. This patient population is at a high risk of both general PICS and the specific conditions associated with post-COVID-19 recovery. To manage PICS and PCS more efficiently, personalized, multidisciplinary, and long-term follow-up visits after hospital discharge are needed to provide a clearer understanding of PICS risk factors, independent of the COVID-19 status.

### Limitations

This study has several limitations. First, the primary limitation is the small number of patients included. The sample size of 18 participants is justified by the distinctive and uncommon nature of the patient cohort—individuals who survived SARS-CoV-2 infection with severe ARDS requiring ECMO therapy. Single-center studies provide a controlled environment that reduces variability and allows for detailed analysis, particularly relevant given the strain placed on healthcare systems during the pandemic. We emphasize the exploratory nature of the study and its statistical analyses in light of the small sample size. Despite these caveats, we believe that this study offers valuable insights into the long-term HRQoL and contributes meaningfully to understanding outcomes and improving future treatment strategies. Given the limited cohort, subgroup or stratified analyses were not performed, as they would lack sufficient statistical power and could risk overinterpretation. Finally, we note that the response rate of 46.2% is higher than in comparable follow-up studies [25]. Second, post-ICU follow-up visits are not yet standard in our clinical practice, and all follow-up assessments were conducted via telephone. Third, although the SF-36 questionnaire is conventionally self-administered, its telephone-based administration has been validated and shown to be reliable and acceptable in large-scale studies (e.g., Watson et al.) [70], including for population-level norms. In our study, a single trained physician conducted all of the interviews using a structured script. Nonetheless, we acknowledge that telephone-based interviews may influence how patients respond (e.g., social desirability bias), and this possibility should be considered when interpreting the findings.

## 5. Conclusions

In our cohort, ICU survivors showed reduced HRQOL scores in physical functioning, general health, role limitation due to physical problems, role limitation due to emotional problems, and social functioning. A longer duration of ECMO was associated with a better HRQOL in the domains of vitality and emotional well-being; however, this finding must be interpreted cautiously in light of possible survivorship bias and the exploratory nature of the study. Our results also emphasize the importance of post-ICU rehabilitation programs and psychological support to mitigate long-term impairments. In conclusion, survivors of critical illness due to COVID-19 ARDS requiring IMV and ECMO therapy exhibit a high prevalence of reduced HRQOL more than 18 months after discharge. Larger-scale, multi-center follow-up studies are needed to validate these findings, explore causal relationships, and inform post-ICU care strategies.

## Figures and Tables

**Table 1:** SF-36 HRQOL > 18 months after ICU discharge. Outcomes compared to non-COVID-19 ECMO patients [38].

SF-36 Subscale	Current Study Population	Turgeon et al. [38]	
	Mean (SD)	Pooled Weighted Mean Scores	*p*
	n = 18	n = 289	
Physical functioning	62.22 [±32.95]	70.31	0.02
Role limitation due to physical problems	37.50 [±48.70]	49.62	0.72
Role limitation due to emotional problems	47.92 [±50.14] ^a^	69.82 ^c^	0.09
Energy/fatigue (vitality)	51.02 [±33.97]	54.46	0.66
Emotional well-being	70.89 [±22.83]	68.8 ^d^	0.54
Social functioning	38.14 [±41.45]	71.42 ^b^	<0.01
Pain	75.14 [±32.95]	70.41	0.82
General health	49.24 [±30.03]	56.47	<0.01

SF-36 = Short-Form Health Survey-36. Subscales by mean [±SD]. ^a^ n = 16; ^b^ n = 222; ^c^ n = 204; ^d^ n = 227.

**Table 2:** Demographic data.

**Demographic**	**Mean (SD); No, %**	**n = 18**
Age, years	52 [±9.5]	
Sex, male (%)	9 (50)	
BMI, kg/m^2^	30 [±5]	
Marital status, married (%)	14 (78)	
Return to work, yes (%)	8 (44)	
Early retirement (%)	3 (17)	
Inability to work at time of the interview (%)	7 (39)	
**ICU Data**	**Mean (SD)**	
SAPS III	66.35 [±7.87]	n = 17
ICU LOS, days	41.6 [±21.6]	
ECMO duration, days	21.4 [±12.2]	
IMV pre-ECMO duration, days	8.93 [±7.96]	
IMV duration, days	25.2 [±11.83]	
Days on sedatives *	31.8 [±13.6]	
Days on benzodiazepines °	19.9 [±10.4]	
Days on neuroleptics ^	20.2 [±11.4]	
Days on NMBA	5.06 [±3.1]	
**Comorbidities**	**no. (%)**	
Underlying pulmonary disease, no. (%)	4 (22)	
Arterial hypertension, no. (%)	10 (56)	
Obesity, no. (%)	8 (44)	
Diabetes mellitus, no. (%)	4 (22)	
Chronic heart disease, no. (%)	1 (6)	
Immunosuppression, no. (%)	1 (6)	
Chronic kidney disease, no. (%)	1 (6)	
Mental or cognitive problems, no. (%)	0 (0)	
No underlying disease, no. (%)	4 (22)	

Metric data are reported as mean [±SD], where n is the number of available observations. BMI = body mass index; ECMO, extracorporeal membrane oxygenation; ICU LOS, intensive care unit length of stay; SAPS, Simplified Acute Physiology Score; IMV, invasive mechanical ventilation; * Clonidine, Dexmedetomidine, Esketamine, Fentanyl, Sufentanil, Hydromorphone, Morphine, Propofol; ° Diazepam, Lorazepam, Midazolam, Oxazepam; ^ Risperidone, Quetiapine; frequent comorbidities according to Attaway et al. [42].

## Data Availability

Supporting data from this study were obtained by emailing the corresponding author. The datasets used and analyzed during the current study are available from the corresponding author on reasonable request.

## Supplementary Materials

The following supporting information can be downloaded at: https://www.mdpi.com/article/10.3390/jcm14103358/s1, Supplemental Table S1. Statistically significant correlations of HRQOL variables with demographic data and ICU-related data. Abbreviations: IMV = invasive mechanical ventilation; ICU = intensive care unit; ECMO = extracorporeal membrane oxygenation; NMBA = neuromuscular blocking agents. Supplemental Table S2. Demographic and ICU data of patients who did not respond to the HRQOL follow-up.

## List of Abbreviations

ARDS	Acute respiratory distress syndrome
BMI	Body mass index
COVID-19	Coronavirus disease 2019
ECMO	Extracorporeal membrane oxygenation
EQ-5D	EuroQOL-5D
HRQOL	Health-related quality of life
ICU	Intensive care unit
IMV	Invasive mechanical ventilation
IQR	Interquartile range
LOS	Length of stay
NMBA	Neuromuscular blocking agent
PCS	Post-acute COVID-19 syndrome
PICS	Post-intensive care syndrome
PTSD	Posttraumatic stress disorder
SAPS III	Simplified Acute Physiology Score III
SARS-CoV-2	Severe acute respiratory syndrome coronavirus 2
SD	Standard deviation
SF-36	36-Item Short-Form Health Survey
