# Supplementary material for: Long-Term Health-Related Quality of Life Following Survival of Acute Respiratory Distress Syndrome and Extracorporeal Membrane Oxygenation Due to COVID-19
Source: J Clin Med. 2025 May 12;14(10):3358. doi: 10.3390/jcm14103358 (PMC12112094; doi:10.3390/jcm14103358)
Supplement: Supplementary file 1 [file jcm-14-03358-s001.zip › jcm-3605293-supplementary.pdf]

Variable 1	Variable 2	P value
Energy/Fatigue	General Health	< 0.001
IMV Duration (days)	IMV Duration pre-ECMO (days)	< 0.001
Role limitation. emotional	Energy/Fatigue	< 0.001
Length of ICU Stay (days)	Sedatives (days)	< 0.001
Physical Functioning	Energy/Fatigue	< 0.001
Physical Functioning	General Health	< 0.001
Benzodiapines (days)	Sedatives (days)	0.001
Role limitation. emotional	General Health	0.001
Role limitation. physical	General Health	0.001
Energy/Fatigue	IMV Duration (days)	0.002
Length of ICU Stay	Benzodiapines (days)	0.002
Role limitation. physical	Role limitation. emotional	0.002
Neuroleptics (days)	Sedatives (days)	0.002
Role limitation. physical	Energy/Fatigue	0.003
Sedatives (days)	ECMO Duration (days)	0.003
Energy/Fatigue	Age	0.003
General Health	Age	0.004
Energy/Fatigue	IMV Duration pre-ECMO (days)	0.005
Physical Functioning	Role limitation. physical	0.006
Physical Functioning	Role limitation. emotional	0.006
General Health	IMV Duration (days)	0.007
General Health	IMV Duration pre-ECMO (days)	0.009
Length of ICU Stay (days)	Neuroleptics (days)	0.010
Physical Functioning	Social Functioning	0.013
Role limitation. physical	Social Functioning	0.016
Emotional Wellbeing	ECMO Duration (days)	0.018
Physical Functioning	IMV Duration pre-ECMO (days)	0.019
Energy/Fatigue	Emotional Wellbeing	0.02
Sex (male)	NMBA (days)	0.024
Emotional Wellbeing	IMV Duration (days)	0.028
Body Mass Index	NMBA (days)	0.035
Energy/Fatigue	ECMO Duration (days)	0.04
Physical Functioning	Body Mass Index	0.048
Physical Functioning	Age	0.048

Supplemental Table S1 Statistically significant correlations of HRQOL variables with demographic data and ICU-related data; IMV=Invasive mechanical ventilation; ICU=intensive care unit; ECMO=extracorporeal membrane oxygenation; NMBA=neuromuscular blocking agents.

Demographic	Mean (SD); No, %	n = 25
Age, years	51 [±11]	
Sex, male (%)	16 (64)	
**ICU Data**	**Mean (SD)**	
ICU LOS, days	38.6 [±17.6]	
ECMO duration, days	19.7 [±13.9]	

Supplemental Table S2 Demographic and ICU data of patient without response to HRQOL follow up.
